# Temperature and Pressure Dependence of the Reaction between Ethyl Radical and Molecular Oxygen: Experiments and Master Equation Simulations

**DOI:** 10.1021/acs.jpca.2c07780

**Published:** 2023-01-25

**Authors:** Timo T. Pekkanen, Raimo S. Timonen, Elli A. Ramu, György Lendvay, Arkke J. Eskola

**Affiliations:** † Department of Chemistry, University of Helsinki, P.O. Box 55 (A.I. Virtasen aukio 1), 00014 Helsinki, Finland; ‡ Institute of Materials and Environmental Chemistry, Research Centre for Natural Sciences, Magyar Tudósok krt. 2, Budapest H-1117, Hungary

## Abstract

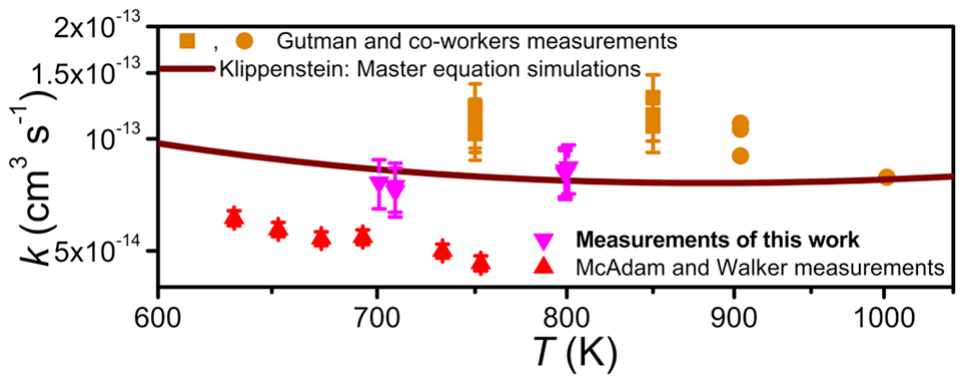

We have used laser-photolysis – photoionization mass-spectrometry to measure the rate coefficient for the reaction between ethyl radical and molecular oxygen as a function of temperature (190–801 K) and pressure (0.2–6 Torr) under pseudo-first-order conditions ([He] ≫ [O_2_] ≫ [C_2_H_5_^•^]). Multiple ethyl precursor, photolysis wavelength, reactor material, and coating combinations were used. We reinvestigated the temperature dependence of the title reaction’s rate coefficient to resolve inconsistencies in existing data. The current results indicate that some literature values for the rate coefficient may indeed be slightly too large. The experimental work was complemented with master equation simulations. We used the current and some previous rate coefficient measurements to optimize the values of key parameters in the master equation model. After optimization, the model was able to reproduce experimental falloff curves and C_2_H_4_ + HO_2_^•^ yields. We then used the model to perform simulations over wide temperature (200–1500 K) and pressure (10^–4^–10^2^ bar) ranges and provide the results in PLOG format to facilitate their use in atmospheric and combustion models.

## Introduction

The reaction between ethyl and molecular oxygen is a prototypical fuel radical + O_2_ reaction, as it contains all the main alkane oxidation reaction channels. However, in practice only the conjugate-alkene-forming channel is kinetically important.^1−5^ High-level computations^6,7^ predict the relative energy of the concerted-elimination transition structure of this channel to be 9–13 kJ mol^–1^ below the energy of the separated reactants, whereas the barrier for the second-most-important channel, QOOH formation, is over 26 kJ mol^–1^ higher in energy.^6^ The conjugate alkene channel forms ethene and hydroperoxyl either by the sequential mechanism,
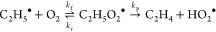
R1or directly through well-skipping (ws),^8^
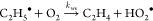
R2As the concerted-elimination transition structure is below the energy of the separated reactants, ethene and hydroperoxyl formation through reaction R2 can already be observed at room temperature if the pressure is low enough.^9−11^ Thus, the overall low-temperature rate coefficient is the sum
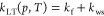
1

An interesting feature of the title reaction, and of R^•^ + O_2_ reactions in general, is the change in temperature and pressure dependence of the observed rate coefficient as the high-temperature regime is entered. The exact temperature ranges of the low- and high-temperature regimes depend on pressure and [O_2_], but they are roughly *T* < 550 K and *T* > 750 K in the present case.^12^ In both limiting regimes, the ethyl concentration decays exponentially, provided that [O_2_] ≫ [C_2_H_5_^•^]. In between these ranges there is a transitional regime in which C_2_H_5_^•^ + O_2_ ⇌ C_2_H_5_O_2_^•^ equilibration is important and C_2_H_5_^•^ decays are double-exponential.^13,14^ The low-temperature rate coefficient exhibits pressure dependence and negative temperature dependence, which is typical for barrierless R^•^ + O_2_ → RO_2_^•^ recombination reactions. As the temperature is increased to above ∼700 K, the C_2_H_5_^•^ + O_2_ ⇌ C_2_H_5_O_2_^•^ equilibrium begins to overwhelmingly favor the reactants (again, depending somewhat on the employed reactant concentration), and single-exponential decays re-emerge, from which a phenomenological rate coefficient can be extracted.^11,15^ In contrast to the situation at low temperatures, the high-temperature rate coefficient is pressure-independent and has a weak, positive temperature dependence. The high-temperature rate coefficient corresponds to the phenomenological reaction
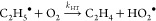
and both the the sequential (reaction R1) and well-skipping (reaction R2) mechanisms contribute to it. High pressures and/or low temperatures favor the sequential mechanism, while the opposite conditions favor the well-skipping mechanism. The phenomenological high-temperature rate coefficient can be expressed in terms of the elementary rate coefficients in reactions R1 and R2 if the pre-equilibrium approximation is made, giving
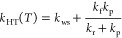
2The reasons why *k*_HT_(*T*) is pressure-independent have been discussed by Miller and co-workers, and the readers are referred to their work.^12,16^ Briefly, at high temperatures the peroxyl adduct reaches what they call its stabilization limit. At and beyond this limit, a significant fraction of the peroxyl adduct’s Boltzmann population is above the energy threshold to form ethene and hydroperoxyl. Thus, collisions not only support relaxation into the RO_2_^•^ well but also repopulate the high energy levels that are depleted by the product channel. Furthermore, at the stabilization limit activating collisions start to become as probable as deactivating ones. A consequence of this is that no long-lived RO_2_^•^ adducts are formed, no matter how high the pressure is.

The master equation (ME) simulations of Miller and co-workers also revealed that the chemically significant eigenvalue (CSE) that corresponds to the overall rate coefficient of the C_2_H_5_^•^ + O_2_ reaction “jumps” from the most negative CSE to the least negative CSE as the high-temperature regime is entered. If only channels R1 and R2 are considered, there are two CSEs, and the low- and high-temperature rate coefficients are given, to a good approximation, by
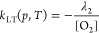
3and
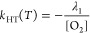
4respectively. In the transitional regime, multiexponential decays are observed, and there is no rate coefficient that can be associated with a single CSE. The location and width of the transitional temperature regime depends on pressure and [O_2_]. At very low pressures it vanishes completely, and there is a seamless transition from *k*_LT_(*p*, *T*) to *k*_HT_(*T*).^16^

**Figure 1:**
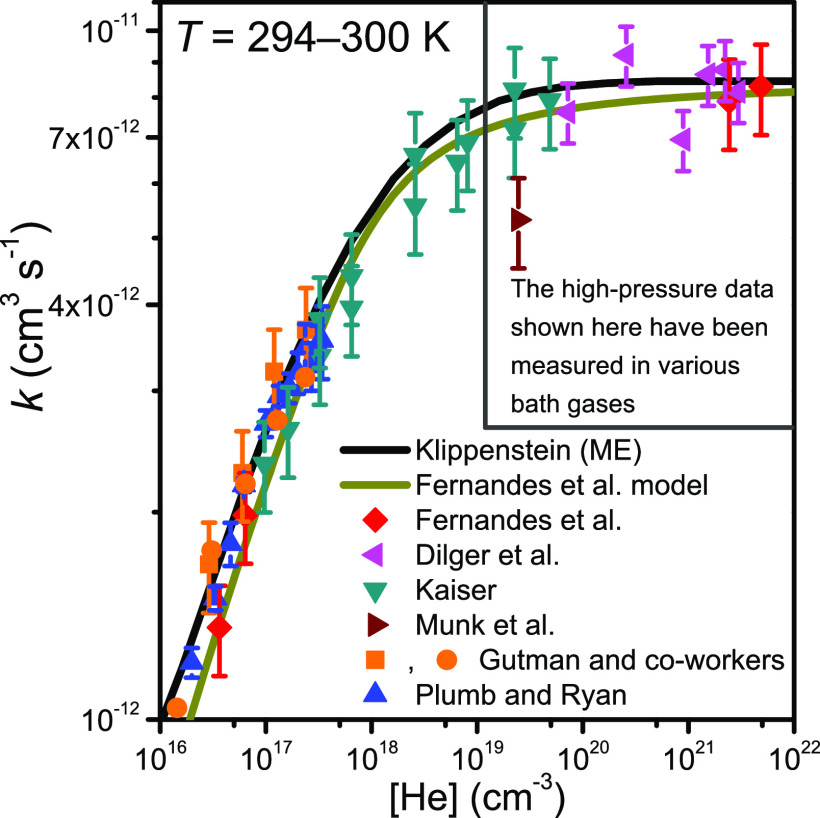
Pressure dependence of the C_2_H_5_^•^ + O_2_ rate coefficient at around room temperature.^6,9,11,15,17−20^

**Figure 2:**
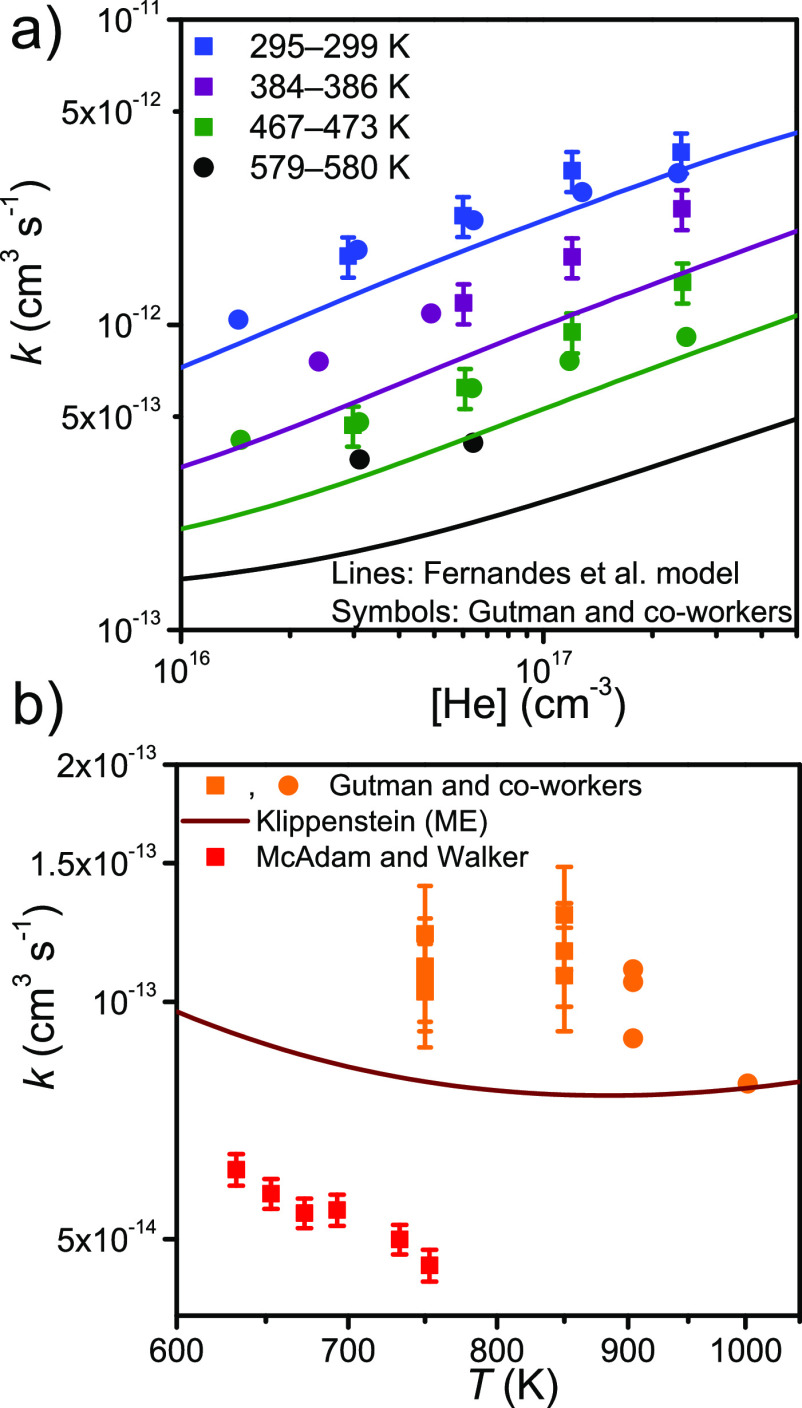
Temperature and pressure dependence of the C_2_H_5_^•^ + O_2_ rate coefficient.^3,6,11,15,20^ Above ∼700 K, the rate coefficient is pressure-independent.

The title reaction has been thoroughly studied with experimental methods at room temperature, and the results are in good agreement with each other.^9,11,15,17−19^ The room-temperature falloff curve is also reproduced by the modeling work of Fernandes et al.^20^ and the ME simulations of Klippenstein.^6^ This is shown in Figure 1. However, above room temperature there is more scatter in the experimental, modeling, and computational results, which is illustrated in Figure 2. The modeling work of Fernandes et al. and the measurements of McAdam and Walker^3^ in the low- and high-temperature regimes, respectively, yield smaller rate coefficient values than the measurements of Gutman and co-workers.^11,15^ Klippenstein’s high-temperature simulations also predict a smaller rate coefficient.

Gutman and co-workers used two different ways to produce ethyl in their experiments: abstraction of a hydrogen from ethane with a chlorine atom or photolysis of bromoethane with 248 nm photons. The Cl^•^-initiated and bromoethane measurements are depicted with circles and squares, respectively, in Figures 1 and 2. The two reaction initiation methods produce consistent results at room temperature, but there is some disagreement (∼30%) at elevated temperatures. Furthermore, the wall rates (i.e., the disappearance rates of ethyl in the absence of added O_2_) reported by Gutman and co-workers are quite high (30–140 s^–1^). We use a very similar experimental setup^21^ in Helsinki and have obtained much smaller wall rates for ethyl (<30 s^–1^) with various reactor material–coating combinations.^22,23^

In this work, we have measured the rate coefficient between ethyl and molecular oxygen over a wide temperature range (190–801 K) and a modest pressure range (0.2–6 Torr). Different ethyl precursor, photolysis wavelength, reactor material, and coating combinations were used to check that consistent results were obtained. One motivation of the present study was to determine whether the results reported by Gutman and co-workers^11,15^ are “too high”, as suggested by Fernandes et al.^20^ In addition to the experimental work, we have performed ME simulations to extrapolate the experimental results to conditions more relevant for atmospheric and combustion chemistry.

## Methods

### Experimental Section

The experimental setup was described in a previous publication,^21^ and only the details relevant to the current work are given here. We performed the experiments in laminar flow reactors made of stainless steel (i.d. = 0.80 or 1.70 cm), Pyrex (i.d. = 1.65 cm), or quartz (0.85 or 1.70 cm). The stainless steel, Pyrex, and quartz reactors were coated with halocarbon wax, polydimethylsiloxane (PDMS), and boric oxide, respectively. A few experiments were also performed with an uncoated quartz reactor. The purpose of the coating is to make the reactor surface as inert as possible to minimize the rate at which ethyl reacts with the reactor wall. Helium bath gas was used, and it always constituted the bulk (>95%) of the flow. Molecular oxygen was always in large excess over the initial ethyl concentration ([O_2_]/[C_2_H_5_^•^] >50) to ensure that pseudo-first-order conditions were realized.

Ethyl radicals were homogeneously produced along the reactor using a pulsed ArF or KrF exciplex laser. The following radical precursors and photolysis reactions were used:
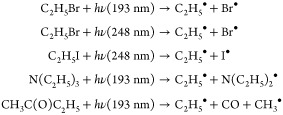
Note that only the ethyl-forming photolysis channels are shown. The radical precursors were degassed by several freeze–pump–thaw cycles before use. The gaseous radical precursor was introduced into the reactor by bubbling helium through temperature-controlled liquid precursor. A portion (3–20%) of the flowing gas mixture was sampled into a vacuum chamber containing a quadrupole mass spectrometer through a small hole on the side of the reactor. Ethyl radicals were prepared for mass spectrometric detection by ionizing them with a chlorine lamp (8.9–9.1 eV). A hydrogen lamp (10.2 eV) was also tested in a single room-temperature experiment. The measurement gave the same value (within experimental uncertainty) for the bimolecular rate coefficient as similar chlorine lamp measurements, although it was seen that the signal did not return to the prephotolysis background. This indicates that a hydrogen lamp is able to dissociatively ionize the peroxyl adduct (C_2_H_5_O_2_^+^ → C_2_H_5_^+^ + O_2_). A CaF_2_ or BaF_2_ window was used with the chlorine lamp, and a MgF_2_ window was used with the hydrogen lamp. The purpose of the window is to cut off radiation higher than that wanted for ionization.

Absorption cross-sections at 193 and 248 nm are known for bromo- and iodoethane at room temperature and can be used to estimate the initial ethyl concentration in our experiments.^24^ The JPL recommended values for bromoethane are 61 × 10^–20^ cm^2^ at 193 nm and 1.1 × 10^–20^ cm^2^ at 248 m. For iodoethane, the absorption cross-section is 95 × 10^–20^ cm^2^ at 248 nm. In the initial ethyl concentration calculations, we assumed that these absorption cross sections are temperature-independent and that the quantum yields for the ethyl-forming channels are unity. Furthermore, we did not account for how much of the laser pulse is cut by the front window (quartz or MgF_2_) of the reactor. Thus, the *absolute* initial radical concentrations we report in this work are only rough upper estimates. However, the values still give a reasonable estimate of the *relative* differences in initial ethyl concentrations, especially at a given temperature.

**Figure 3:**
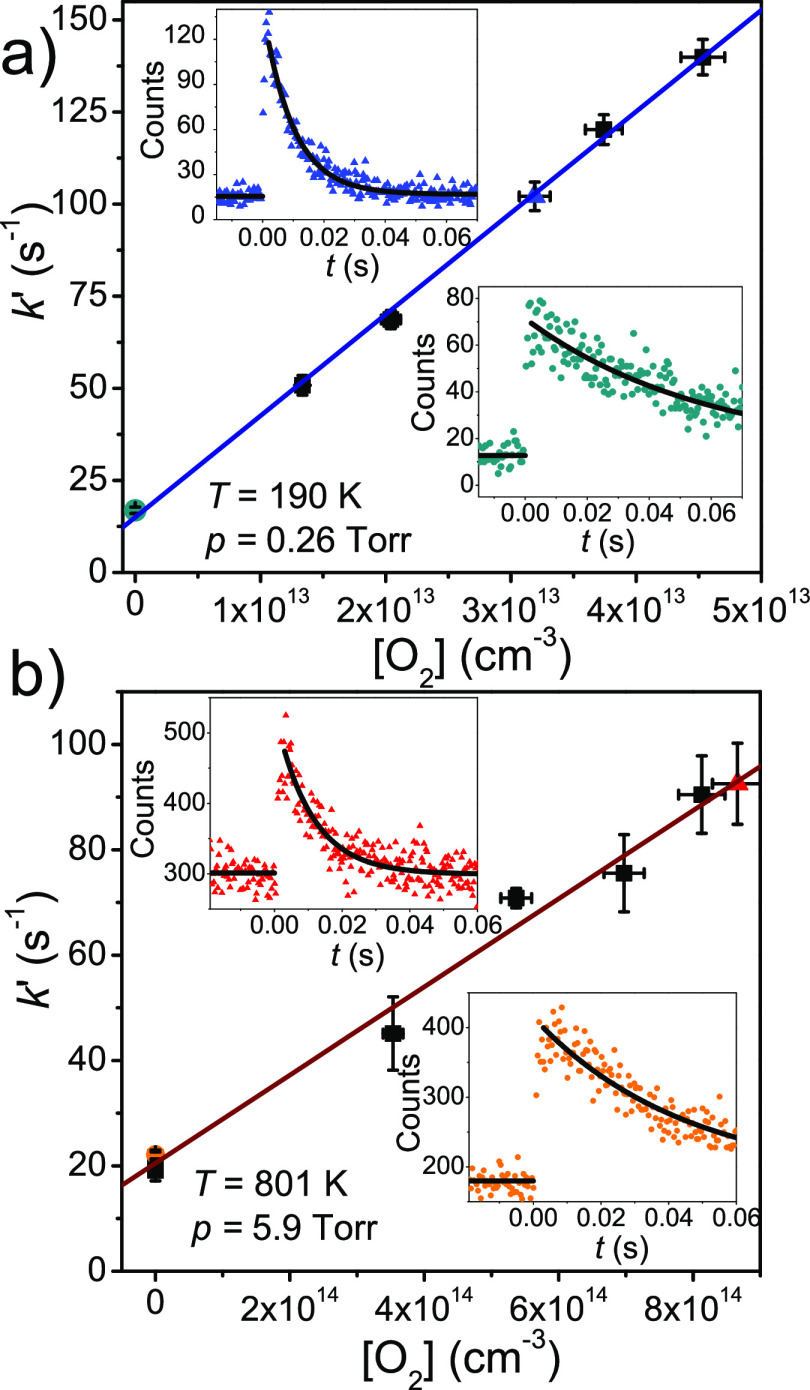
Examples of bimolecular plots at (a) 190 K and 0.26 Torr and (b) 801 K and 5.9 Torr. The ethyl traces shown in the bottom right and top left corners were measured in the absence and presence of O_2_, respectively. The colored symbols correspond to the similarly colored traces.

We started each bimolecular rate coefficient measurement by determining the wall rate *k*_w_, which describes the first-order-decay of ethyl in the absence of added O_2_ and is mainly due to the reaction between ethyl and the reactor wall. The self-reaction of ethyl and the reaction between ethyl and the precursor also contribute to *k*_w_, but these are minimized by using low radical and precursor concentrations. The wall rate measurement was repeated at the end to ensure that it had remained approximately constant. We determined *k*_w_ by monitoring the decay of ethyl in real time and fitting the function
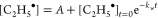
5to the obtained trace. Here *A* is the signal background and *t* is time. Note that although the radical concentration is used in the equation, in fact it denotes the signal that is directly proportional to it. Absolute concentrations are not needed to determine the decay constant. After the initial wall rate measurement, a known concentration of O_2_ was added to the reactor, and the decay of ethyl was again monitored. A single-exponential function
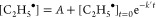
6was fitted to the trace to obtain the pseudo-first-order rate coefficient *k*′, which is related to the bimolecular rate coefficient (*k*) of the title reaction by
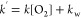
7The pseudo-first-order rate coefficient was typically measured at three to eight different O_2_ concentrations. When these were plotted as a function of [O_2_], the slope of a straight line fitted to the points gave *k*. The intercept with *y*-axis gave an estimate for *k*_w_, which should agree with the directly measured values if the experiments have been correctly performed. We report both values. Examples of bimolecular plots are given in Figure 3. We estimate that the overall uncertainty in the bimolecular rate coefficient measurements is ±15%. This arises mainly from uncertainties in [O_2_], which in turn results from uncertainties in measured flow rates.

### Master Equation

We used the MESMER 6.1 program in our ME simulations.^25^ To simplify the simulations, we included only channels R1 and R2 in the model. As mentioned in the Introduction, the other channels are of minor relevance and are not needed to interpret the experimental data. Klippenstein recently investigated the potential energy surface of the title reaction with high-level methods, and here we use his stationary point geometries and harmonic frequencies.^6^ The methyl group rotation in ethyl radical and in the loose C_2_H_5_^•^ + O_2_ → C_2_H_5_O_2_^•^ recombination transition state (referred to as the “loose TS” from here on) was treated as a classical free rotor with a rotational constant of 15.07 cm^–1^. The MN15/Def2TZVP method^26,27^ predicts the rotational barrier in ethyl to be as low as about 0.2 kJ mol^–1^, so the classical free-rotor approximation should be a good one. We also used the MN15/Def2TZVP method to compute torsional potentials for the peroxyl adduct. To treat the coupling between the hindered rotors and external rotation in the adduct, we applied the method of Gang et al. implemented in MESMER (the current implementation does not explicitly account for potential coupling).^28^ This method is fully classical, so to avoid double-counting of zero-point energy (ZPE) contributions, we subtracted from the relative energy of the peroxyl adduct the hindered rotors’ ZPEs (∼1.96 kJ mol^–1^). These were obtained using a one-dimensional quantum-mechanical hindered-rotor treatment.

For the reaction over the concerted-elimination transition state, we used conventional RRKM theory to compute the microcanonical rate coefficient. Eckart tunneling corrections were included. Conventional RRKM theory cannot be used for the barrierless recombination reaction, as there is no saddle point. Instead, we used the RRKM expression together with the state sum from Klippenstein^6^ for the loose TS to compute the microcanonical rate coefficient. He obtained the energy-dependent and *J*-averaged state sum using variable reaction coordinate transition state theory (VRC-TST).^29^ Klippenstein multiplied the state sum by a factor of 0.85 before performing ME simulations. We suspect that the factor was applied to approximately correct for recrossing effects, and the value was chosen on the basis that then the experimental high-pressure rate coefficient was reproduced at room temperature. Be that as it may, transition state theory often overestimates rate coefficients by 10–20% even when the dividing surface location is variationally optimized, so this correction factor is perfectly reasonable. We chose to apply the same correction.

For comparison purposes, we also used the inverse Laplace transform (ILT) technique implemented in MESMER to obtain the state sum for the loose TS.^30,31^ The function that is transformed is the modified Arrhenius expression for the high-pressure (canonical) recombination rate coefficient:
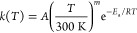
8While the modified Arrhenius parameters are generally not known for a given reaction, optimal values for them can be obtained by fitting against experimental data. For barrierless reactions, *E*_a_ is usually set to zero, and this was also done in this work.

Collisional energy transfer was treated with the standard exponential-down model,
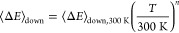
9where ⟨Δ*E*⟩_down,300K_ is the average energy transferred downward in collisions at 300 K and *n* accounts for the temperature dependence of the energy transfer process. These parameters can be similarly (and simultaneously) optimized against experimental data with the modified Arrhenius parameters. Lennard-Jones (LJ) interaction potentials were used to calculate collision frequencies. The following values were used:
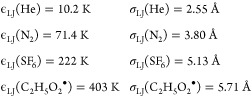
The values for the bath gases were obtained from the literature.^32^ For the peroxyl adduct, we used the LJ parameters of ethaneperoxol. These were estimated using the online resources of Cantherm (Joback method).^33^

The overall rotational symmetry numbers for C_2_H_5_^•^, O_2_, C_2_H_5_O_2_^•^, and the concerted-elimination transition state are 6, 2, 3, and 1, respectively. The corresponding electronic partition functions are 2, 3, 2, and 2. For the loose TS, the overall rotational symmetry number and electronic partition function are 12 and 2, respectively. The energy grain size used in the simulations was 40 cm^–1^, and the cutoff energy was set to 25*k*_B_*T* above the highest-energy stationary point.

## Results and Discussion

### Comparison with Previous Measurements

**Figure 4:**
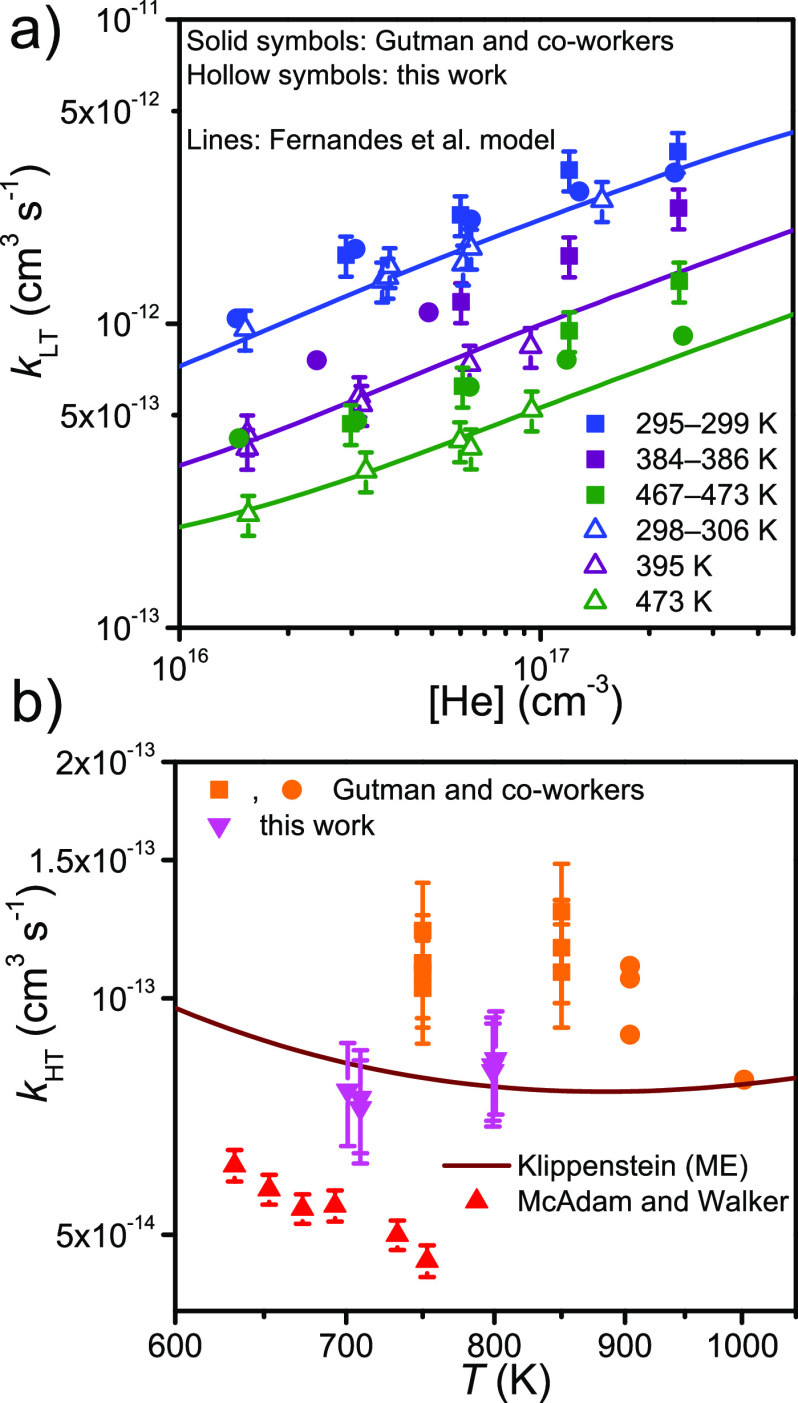
Temperature and pressure dependence of the rate coefficient between ethyl and molecular oxygen in the (a) low- and (b) high-temperature regimes.^3,6,11,15,20^ Above ∼700 K, the rate coefficient is pressure-independent.

The results and conditions of our bimolecular rate coefficient measurements are tabulated in Table 1. As can be seen, consistent results have been obtained with many different ethyl precursor, photolysis wavelength, reactor material, and coating combinations. The measurements with an uncoated quartz reactor at 710 K are an exception. Furthermore, the wall rates we measure for ethyl are much smaller than those reported by Gutman and co-workers.^11,15^ It is also evident that our results are independent of the initial radical concentration. Thus, we are confident that the precursor and initial radical concentrations are low enough that secondary chemistry is suppressed. In Figure 4 we compare the current results to those of Gutman and co-workers,^11,15^ Fernandes et al.,^20^ McAdam and Walker,^3^ and Klippenstein.^6^ For the low-temperature rate coefficient, the current results are in good agreement with the model of Fernandes et al. but consistently smaller than the measurements by Gutman and co-workers. The disagreement is about 20% at 300 K and increases to 40–50% at 470 K. In the high-temperature regime, the current results agree to within experimental uncertainty with the ME prediction of Klippenstein. Again, our rate coefficient measurements produce values that are about 40% smaller than those by Gutman and co-workers.

**Table 1:** Experimental Conditions and Results of C_2_H_5_^•^ + O_2_ Bimolecular Rate Coefficient Measurements

*T* (K)	*p*_He_ (Torr)	[He] (10^16^ cm^–3^)	[C_2_H_5_]_0_ (10^10^ cm^–3^)^a^	[O_2_] (10^13^ cm^–3^)	*k*′ (s^–1^)^b^	*k*_w_ (s^–1^)^c^	*k*_w_ (s^–1^)^d^	*k* (10^–14^ cm^3^ s^–1^)^e^
190^f^^,^^l^^,^^q^	0.26	1.33	12	1.34–4.53	50.8–140	16.9 ± 0.9	15.0 ± 1.6	275 ± 5
190^f^^,^^l^^,^^q^	0.69	3.53	7.5	1.02–3.63	50.2–141	21.0 ± 1.2	19.5 ± 1.4	332 ± 7
190^f^^,^^l^^,^^q^	1.22	6.21	11	1.07–3.75	65.7–177	19.5 ± 1.8	19.0 ± 4.3	404 ± 19
190^g^^,^^m^^,^^r^	3.67	14.4	–	0.96–3.73	77.0–246	22.8 ± 1.3	26.9 ± 9.1	569 ± 38
								
201^f^^,^^l^^,^^q^	0.28	1.34	9.3	1.93–5.92	46.4–151	13.7 ± 1.3	9.76 ± 3.97	226 ± 12
202^f^^,^^l^^,^^q^	0.75	3.56	6.6	1.10–3.42	38.4–107	11.0 ± 1.4	10.4 ± 1.8	284 ± 9
201^f^^,^^l^^,^^q^	1.28	6.16	11	0.95–4.33	50.1–167	19.2 ± 1.4	20.3 ± 3.5	350 ± 12
								
221^f^^,^^l^^,^^q^	0.32	1.38	7.6	3.12–9.12	64.8–184	4.85 ± 1.81	3.96 ± 6.51	203 ± 11
221^f^^,^^l^^,^^q^	0.84	3.65	48	0.85–2.59	26.2–68.8	4.52 ± 1.23	4.65 ± 0.63	253 ± 4
221^f^^,^^l^^,^^q^	1.47	6.42	43	1.34–4.24	43.1–126	9.61 ± 0.84	12.15 ± 9.66	317 ± 39
								
243^f^^,^^l^^,^^q^	0.18	0.719	12	5.50–14.2	90.3–201	8.25 ± 0.89	12.6 ± 3.6	136 ± 5
243^f^^,^^l^^,^^q^	0.38	1.49	13	1.66–4.74	25.8–80.8	4.90 ± 1.82	3.27 ± 4.50	170 ± 15
243^f^^,^^l^^,^^q^	0.91	3.63	27	2.40–5.09	64.0–127	4.28 ± 1.00	3.50 ± 5.25	233 ± 16
241^f^^,^^l^^,^^q^	1.59	6.38	–	1.49–6.75	54.1–202	9.67 ± 0.66	10.0 ± 2.0	285 ± 5
243^g^^,^^m^^,^^r^	3.67	14.6	–	0.47–3.36	30.1–145	9.22 ± 1.05	13.1 ± 3.4	404 ± 18
								
266^f^^,^^l^^,^^q^	0.19	0.694	3.1	5.16–13.6	56.9–133	6.45 ± 0.97	8.63 ± 3.71	93.0 ± 4.3
266^f^^,^^l^^,^^q^	0.20	0.715	9.8	4.60–15.9	52.0–175	5.11 ± 0.89	3.67 ± 1.37	107 ± 1
267^f^^,^^l^^,^^q^	0.20	0.715	1.4	4.73–21.4	48.0–197	8.04 ± 0.87	8.14 ± 1.81	88.4 ± 1.4
266^f^^,^^l^^,^^q^	0.42	1.51	37	3.37–8.12	54.7–106	8.09 ± 0.60	9.63 ± 2.62	120 ± 5
266^f^^,^^l^^,^^q^	0.99	3.61	70	2.62–7.52	56.6–145	8.63 ± 0.72	8.26 ± 1.26	181 ± 3
266^f^^,^^l^^,^^q^	1.00	3.62	35	1.85–7.30	48.1–148	9.68 ± 0.60	11.5 ± 2.4	188 ± 6
266^f^^,^^l^^,^^q^	1.00	3.62	35	0.91–6.24	27.9–125	8.80 ± 0.49	8.35 ± 2.62	180 ± 7
266^f^^,^^l^^,^^q^	1.77	6.42	77	1.99–5.88	49.9–125	5.48 ± 0.72	6.77 ± 1.74	208 ± 5
								
298^h^^,^^l^^,^^q^	0.21	0.691	3.9	4.77–11.5	43.7–108	3.73 ± 0.87	2.26 ± 2.07	90.5 ± 2.8
298^f^^,^^l^^,^^q^	0.22	0.716	54	9.72–22.5	96.2–203	9.94 ± 0.99	9.93 ± 6.02	84.1 ± 4.2
298^f^^,^^l^^,^^s^	0.22	0.720	41	8.06–22.2	72.7–200	16.2 ± 0.7	20.0 ± 3.4	84.5 ± 2.8
298^f^^,^^l^^,^^q^	0.47	1.52	31	5.07–11.1	57.8–116	8.93 ± 0.75	9.30 ± 3.02	95.9 ± 3.9
298^f^^,^^l^^,^^q^	1.12	3.64	33	1.17–9.40	25.0–135	5.93 ± 0.74	6.59 ± 1.53	138 ± 3
302^f^^,^^l^^,^^q^	1.18	3.77	32	2.24–8.94	40.0–133	7.79 ± 0.62	9.02 ± 1.51	142 ± 3
298^i^^,^^l^^,^^q^	1.18	3.82	–	2.61–11.6	57.3–198	15.7 ± 1.6	19.6 ± 4.7	154 ± 8
306^j^^,^^m^^,^^q^	1.93	6.10	50	4.57–15.8	73.1–297	4.40 ± 1.10	1.94 ± 4.92	157 ± 5
298^f^^,^^n^^,^^q^	1.93	6.24	–	2.95–7.16	57.5–131	3.56 ± 0.50	3.40 ± 1.24	178 ± 3
298^f^^,^^l^^,^^q^	1.98	6.42	50	1.21–7.37	32.8–147	7.83 ± 0.65	8.70 ± 4.57	177 ± 11
298^g^^,^^m^^,^^r^	4.57	14.8	–	2.25–6.65	65.3–178	9.69 ± 1.05	9.45 ± 0.91	254 ± 2
								
333^f^^,^^l^^,^^q^	0.24	0.704	70	8.40–24.2	70.3–166	17.4 ± 0.7	19.1 ± 2.1	62.1 ± 1.4
333^f^^,^^l^^,^^q^	0.54	1.55	32	3.60–14.5	30.3–117	5.90 ± 0.89	1.45 ± 5.35	75.8 ± 6.3
336^f^^,^^n^^,^^q^	1.26	3.62	–	3.56–13.0	33.9–131	1.79 ± 0.67	1.07 ± 1.38	101 ± 2
336^f^^,^^l^^,^^q^	1.28	3.66	62	3.98–11.9	41.9–123	4.32 ± 0.81	4.24 ± 1.39	101 ± 2
336^f^^,^^l^^,^^q^	2.18	6.26	44	4.19–10.4	59.7–152	3.79 ± 1.10	2.95 ± 3.92	130 ± 6
336^f^^,^^l^^,^^q^	2.22	6.37	–	3.45–10.0	46.4–136	1.20 ± 0.50	0.81 ± 1.37	131 ± 2
								
363^f^^,^^l^^,^^q^	0.27	0.729	50	7.76–16.5	47.8–94.4	9.18 ± 0.85	8.75 ± 0.96	52.1 ± 1.4
363^f^^,^^l^^,^^q^	0.58	1.54	30	2.91–14.2	18.7–78.8	4.72 ± 1.00	5.56 ± 2.31	53.1 ± 2.6
363^f^^,^^l^^,^^q^	1.45	3.86	–	2.52–12.8	19.2–104	3.12 ± 0.91	3.79 ± 0.97	74.8 ± 1.4
363^g^^,^^m^^,^^r^	2.28	6.08	79	2.16–10.7	33.5–122	11.7 ± 1.0	10.6 ± 1.9	104 ± 3
363^f^^,^^l^^,^^q^	2.38	6.33	64	2.99–7.97	32.9–83.5	3.00 ± 0.93	2.59 ± 0.92	102 ± 2
363^g^^,^^m^^,^^r^	4.03	10.7	29	2.70–6.82	55.6–104	17.5 ± 0.7	19.8 ± 4.5	132 ± 10
363^g^^,^^m^^,^^r^	5.57	14.8	43	2.32–4.85	50.8–87.9	15.4 ± 0.7	15.5 ± 1.1	152 ± 3
								
395^h^^,^^l^^,^^q^	0.36	0.900	7.8	8.73–20.1	35.9–76.2	4.74 ± 1.01	4.48 ± 1.35	36.0 ± 1.1
395^h^^,^^l^^,^^q^	0.63	1.54	7.5	8.14–22.3	37.5–98.1	2.89 ± 0.82	2.26 ± 1.68	43.4 ± 1.1
395^k^^,^^m^^,^^q^	0.63	1.54	61	4.25–15.9	25.4–61.9	7.78 ± 0.99	7.79 ± 1.32	38.9 ± 1.4
395^k^^,^^m^^,^^q^	1.29	3.15	–	3.15–11.6	29.9–77.7	10.9 ± 0.6	10.9 ± 0.4	57.9 ± 0.6
395^h^^,^^l^^,^^q^	1.31	3.19	5.0	5.61–11.7	32.5–96.8	2.91 ± 0.82	2.60 ± 0.78	54.2 ± 0.7
395^h^^,^^l^^,^^q^	2.61	6.36	8.6	3.43–13.4	27.2–99.5	2.55 ± 0.73	3.28 ± 1.53	73.5 ± 1.8
395^j^^,^^m^^,^^q^	3.84	9.39	14	6.45–25.9	59.2–225	6.80 ± 0.77	7.73 ± 2.18	84.1 ± 1.6
								
430^h^^,^^l^^,^^q^	0.41	0.909	7.8	9.88–25.9	33.2–90.8	4.45 ± 1.05	3.04 ± 1.82	33.1 ± 1.3
430^h^^,^^l^^,^^q^	0.69	1.54	8.8	10.2–23.3	35.7–82.9	3.96 ± 0.79	2.66 ± 1.30	34.1 ± 0.8
430^h^^,^^l^^,^^q^	1.43	3.21	8.7	7.62–20.1	35.8–90.5	4.97 ± 0.89	4.61 ± 0.91	42.6 ± 0.7
430^h^^,^^l^^,^^q^	2.45	5.49	10	5.48–13.6	36.4–81.6	5.05 ± 0.72	5.23 ± 0.49	55.8 ± 0.5
								
473^h^^,^^l^^,^^q^	0.45	0.910	8.4	8.58–29.9	22.0–70.0	3.82 ± 0.82	3.47 ± 0.80	22.4 ± 0.5
473^h^^,^^l^^,^^q^	0.76	1.55	10	6.72–34.3	21.4–85.4	4.05 ± 0.86	4.43 ± 1.03	23.6 ± 0.5
473^h^^,^^l^^,^^q^	1.61	3.28	7.8	10.2–22.4	33.8–76.3	3.20 ± 0.82	1.96 ± 1.36	32.8 ± 0.9
473^j^^,^^m^^,^^q^	2.93	5.98	13	10.2–24.6	48.6–108	7.18 ± 0.79	7.65 ± 1.45	41.2 ± 1.0
473^h^^,^^l^^,^^q^	3.14	6.41	9.4	4.71–27.3	21.5–109	3.35 ± 0.87	3.92 ± 0.93	39.2 ± 0.7
474^j^^,^^m^^,^^q^	4.64	9.45	14	9.55–27.4	55.1–151	6.95 ± 0.77	6.41 ± 0.77	52.0 ± 0.5
								
526^h^^,^^l^^,^^q^	0.23	0.414	9.3	8.60–42.0	29.8–85.9	10.4 ± 0.9	12.4 ± 1.9	18.2 ± 0.7
526^h^^,^^l^^,^^q^	0.47	0.861	18	11.2–23.5	34.7–57.4	9.77 ± 1.12	10.3 ± 2.2	19.8 ± 1.4
526^h^^,^^l^^,^^q^	0.81	1.49	14	10.8–51.0	34.4–116	12.2 ± 0.8	13.3 ± 1.5	20.6 ± 0.6
526^h^^,^^l^^,^^q^	1.71	3.14	13	12.4–41.2	42.6–131	10.7 ± 0.9	8.74 ± 4.51	28.2 ± 1.7
526^h^^,^^l^^,^^q^	3.50	6.43	13	14.0–39.5	59.7–139	13.6 ± 1.0	13.3 ± 4.0	32.4 ± 1.6
								
543^h^^,^^l^^,^^q^	0.23	0.402	10	11.7–39.8	34.3–89.2	11.8 ± 1.0	11.4 ± 1.5	20.0 ± 0.6
543^h^^,^^l^^,^^q^	0.49	0.873	17	13.3–33.5	36.1–79.6	9.05 ± 1.19	8.49 ± 1.60	21.0 ± 0.8
543^h^^,^^l^^,^^q^	0.87	1.54	12	14.9–42.3	43.7–98.9	12.7 ± 0.9	13.6 ± 3.0	21.1 ± 1.1
543^i^^,^^l^^,^^q^	0.98	1.73	–	18.8–47.0	70.1–132	23.2 ± 1.4	22.9 ± 5.5	24.2 ± 1.9
543^h^^,^^l^^,^^q^	1.81	3.21	13	10.2–37.9	35.3–118	14.9 ± 1.2	12.9 ± 2.3	26.8 ± 1.1
541^j^^,^^o^^,^^q^	2.86	5.11	14	16.4–44.4	69.4–155	12.0 ± 1.40	12.9 ± 3.1	31.0 ± 1.2
543^h^^,^^l^^,^^q^	3.60	6.39	15	11.2–40.7	49.9–156	9.65 ± 0.93	10.4 ± 1.4	36.3 ± 0.6
543^j^^,^^m^^,^^q^	3.60	6.40	67	8.38–34.6	27.4–94.7	4.56 ± 0.97	4.97 ± 1.26	26.6 ± 0.7
544^j^^,^^o^^,^^q^	3.63	6.45	17	9.27–37.2	31.9–122	10.1 ± 1.23	8.17 ± 2.11	30.2 ± 1.0
543^j^^,^^m^^,^^q^	5.43	9.50	9.1	10.9–23.3	37.7–82.1	2.64 ± 0.47	2.37 ± 1.32	34.2 ± 1.0
								
701^j^^,^^p^^,^^q^	3.93	5.42	–	38.1–103	39.9–88.3	10.3 ± 0.5	10.8 ± 1.0	7.63 ± 0.18
709^j^^,^^o^^,^^q^	2.88	3.92	13	19.8–119	26.4–94.9	9.20 ± 1.05	11.2 ± 2.3	7.47 ± 0.35
709^j^^,^^o^^,^^q^	4.97	6.77	19	27.6–99.0	33.5–83.5	10.7 ± 1.7	11.9 ± 1.7	7.25 ± 0.29
799^j^^,^^p^^,^^q^	3.00	3.63	–	28.9–115	39.9–112	17.9 ± 0.4	16.2 ± 2.2	8.08 ± 0.35
799^j^^,^^p^^,^^q^	3.00	3.63	–	36.7–111	42.1–99.8	12.0 ± 0.8	12.3 ± 2.5	8.22 ± 0.39
801^j^^,^^p^^,^^q^	5.94	7.15	–	35.4–86.6	45.1–92.5	21.0 ± 1.3	20.5 ± 1.9	8.37 ± 0.36
Unreliable								
710^i^^,^^l^^,^^q^	1.39	1.89	–	12.9–58.8	44.2–126	20.0 ± 1.4	20.5 ± 2.3	17.5 ± 0.7
710^i^^,^^l^^,^^q^	2.15	2.96	57	34.2–96.5	71.7–166	19.9 ± 1.8	19.1 ± 2.5	15.2 ± 0.5
710^i^^,^^l^^,^^q^	2.21	3.00	–	17.4–49.5	46.1–120	16.0 ± 1.9	15.3 ± 4.2	22.2 ± 1.5

a A rough estimate for the initial (*t* = 0) radical concentration (see text for details).

b The pseudo-first-order rate coefficient *k*′ = *k*[O_2_] + *k*_w_.

c Average of measured wall rates. The stated uncertainty is the average standard error (1σ) of the fits. The wall rate is the first-order decay rate of the radical in the absence of added oxygen.

d Wall rate determined from the linear-fit *y*-axis intercept of the bimolecular plot. The stated uncertainty is the standard error (1σ) of the fit.

e Experimentally determined bimolecular rate coefficient (slope of the bimolecular plot). The stated uncertainty is the standard error (1σ) of the linear fit. The estimated overall uncertainty is ±15%.

f Reactor: i.d. = 1.7 cm, stainless steel, halocarbon wax coating.

g Reactor: i.d. = 0.80 cm, stainless steel, halocarbon wax coating.

h Reactor: i.d. = 1.65 cm, quartz, boric oxide coating.

i Reactor: i.d. = 1.65 cm, uncoated quartz.

j Reactor: i.d. = 0.85 cm, quartz, boric oxide coating.

k Reactor: i.d. = 1.65 cm, Pyrex, polydimethylsiloxane coating.

l The radical precursor was C_2_H_5_Br. An ArF laser (193 nm) was used for photolysis.

m The radical precursor was C_2_H_5_I. A KrF laser (248 nm) was used for photolysis.

n The radical precursor was N(C_2_H_5_)_3_. An ArF laser (193 nm) was used for photolysis.

o The radical precursor was C_2_H_5_Br. A KrF laser (248 nm) was used for photolysis.

p The radical precursor was CH_3_C(O)CH_2_CH_3_. An ArF laser (193 nm) was used for photolysis.

q Detection: Cl/CaF_2_.

r Detection: Cl/BaF_2_.

s Detection: H/MgF_2_.

Gutman and co-workers used an uncoated quartz reactor in their measurements. We also performed a few experiments with an uncoated quartz reactor, and consistent results were obtained in the low-temperature regime (*T* < 550 K). However, at ∼700 K we found that the rate coefficient measurements with an uncoated quartz reactor gave larger values than with boric oxide coating. Furthermore, we found it difficult to reproduce the former measurements. The end of Table 1 shows the scatter in the values (15–22 × 10^–14^ cm^3^ s^–1^). Because the boric oxide coating measurements are internally more consistent and reproducible, we deem them to be more reliable. We do not know the reason uncoated quartz measurements gave higher values, but the answer may lie in surface chemistry.

The high-temperature rate coefficient measurements in this work are about 30% larger than the measurements of McAdam and Walker.^3^ Given the indirect way they produce ethyl and measure the rate coefficient, the agreement is remarkably good. They did not measure the rate coefficient directly but rather determined the ratio *k*_HT_/*k*_3_, where *k*_3_ is for the reaction
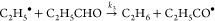
R3Any errors in the Arrhenius parameters of this reaction will affect their results. Note that they did not use the raw Arrhenius parameters available for this reaction^34,35^ but instead corrected those values based on kinetic data available for analogous reactions. Furthermore, the raw Arrhenius parameters for *k*_3_ were themselves obtained from complicated reaction schemes. Thus, the small difference between the current and McAdam–Walker results may well be due to uncertainties in the Arrhenius parameters of reaction R3.

### Parameter Optimization

**Figure 5:**
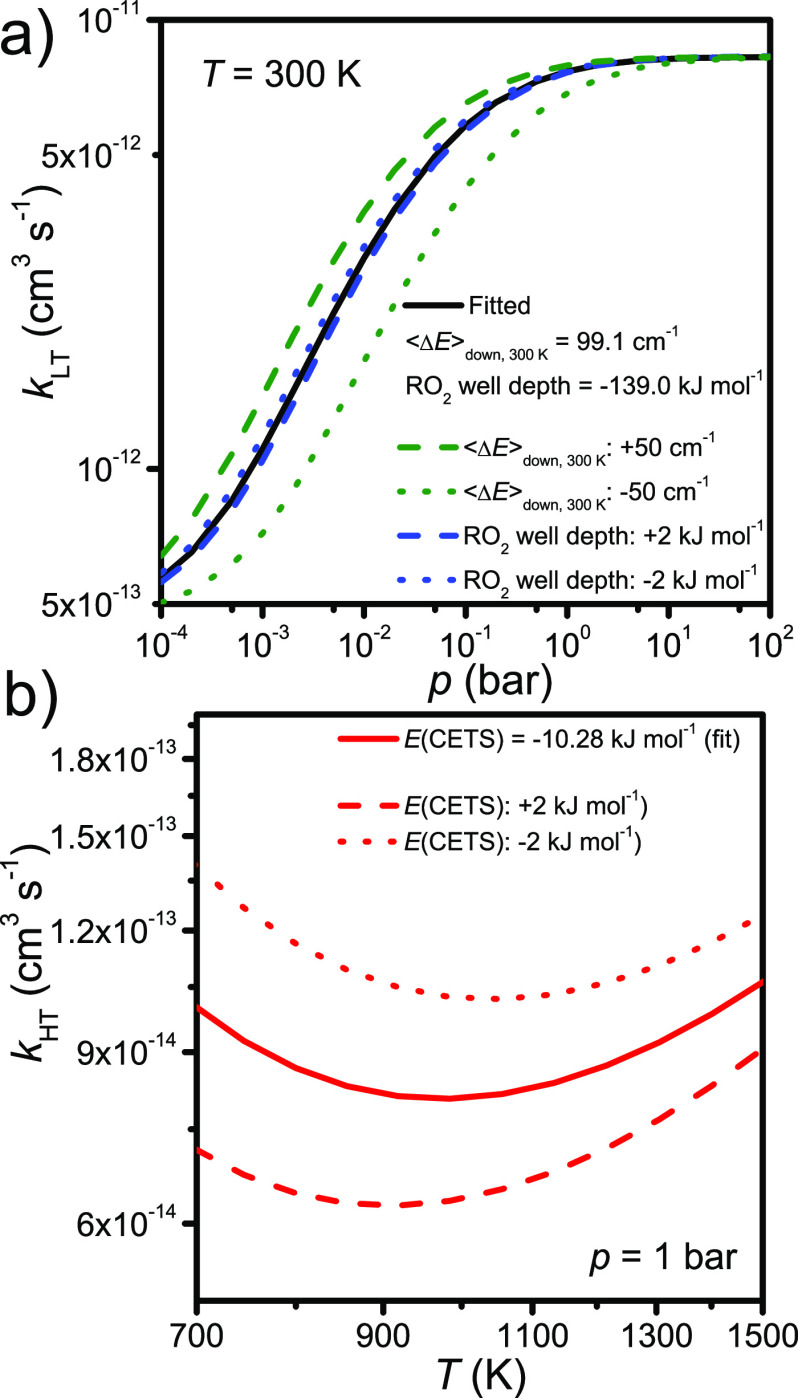
Sensitivity of (a) the low-temperature rate coefficient to the RO_2_^•^ well depth and ⟨Δ*E*⟩_down,300K_ and (b) the high-temperature rate coefficient to the relative energy of the concerted-elimination transition state (CETS).

To optimize the parameters in our ME model, we used the current results together with the kinetic data from Plumb and Ryan,^9^ Kaiser et al.,^17^ Fernandes et al.,^20^ Dilger et al.,^19^ and Knyazev and Slagle.^14^ The high-pressure results from Munk et al.^18^ appear to be outlier data and were not included in the fits. The relative rate results of Kaiser et al. were recalibrated with the most recent rate coefficient data for the reaction^22^
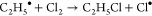
Most of these experiments were performed in helium bath gas, but some of the high-pressure measurements employed other bath gases. We assumed that these latter results are sufficiently close to the high-pressure limit that they can be included in our helium bath gas fits (the results of the fits validated this assumption). We performed two separate fits. In one fit we used the state sum from Klippenstein^6^ for the loose TS (we will call this *N*_VRC-TST_-fit for short). In the other we obtained the state sum using the ILT technique (ILT-fit). The parameters chosen for optimization were the collisional energy transfer parameters (⟨Δ*E*⟩_down,300K_, *n*), the RO_2_^•^ well depth, and the relative energy of the concerted-elimination transition state (CETS). In the ILT-fit the modified Arrhenius parameters (*A*, *m*) of the high-pressure recombination rate coefficient were also optimized. The results of the fits are given in Table 2. The low-temperature rate coefficient is insensitive to the properties of the CETS but sensitive to the collisional energy transfer parameters, the properties of the loose TS, and to a lesser degree to the RO_2_^•^ well depth. The high-temperature rate coefficient, in contrast, is sensitive only to the properties of the CETS. Figure 5 displays how much the low- and high-temperature rate coefficients change as some of these parameters are altered.

**Table 2:** Optimized Master Equation Model Parameters^a^

fit	⟨Δ*E*⟩_down,300K_ (cm^–1^)	*n*	RO_2_^•^ well depth (kJ mol^–1^)	CETS (kJ mol^–1^)	*A* (10^–12^ cm^3^ s^–1^)	*m*
*N*_VRC-TST_(He)	99.1 ± 6.1	1.05 ± 0.08	–139.0 ± 1.1	–10.28 ± 0.23	–	–
ILT	84.1 ± 12.4	1.24 ± 0.20	–139.0 ± 1.0	–10.37 ± 0.20	7.84 ± 0.31	–1.45 ± 0.32
*N*_VRC-TST_(N_2_)	146 ± 8	(1.05)	(−139.0)	(−10.28)	–	–
*N*_VRC-TST_(SF_6_)	384 ± 132	(1.05)	(−139.0)	(−10.28)	–	–

a CETS stands for concerted-elimination transition state. See the text for details about the different fitting schemes. The stated uncertainties are 1σ. In the *N*_VRC-TST_(N_2_) and *N*_VRC-TST_(SF_6_) fits, only ⟨Δ*E*⟩_down,300K_ was optimized; the other parameters were fixed at the values obtained from the *N*_VRC-TST_(He) fit.

The *N*_VRC-TST_- and ILT-fits return remarkably similar values for the RO_2_^•^ well depth and the relative energy of the CETS. We presume the reason for this is that the high-temperature rate coefficient is sensitive to these energies but not to the details of the initial recombination reaction. As demonstrated by Miller and Klippenstein,^16^ the high-temperature kinetics is mainly determined by the properties of the CETS. The ab initio results of Wilke et al.^7^ and Klippenstein^6^ for the well depth are in very good agreement (−139.0 and −137.1 kJ mol^–1^, respectively). The values they report for CETS are −12.47 and −9.54 kJ mol^–1^, respectively. The energy we obtained is between those two values.

**Figure 6:**
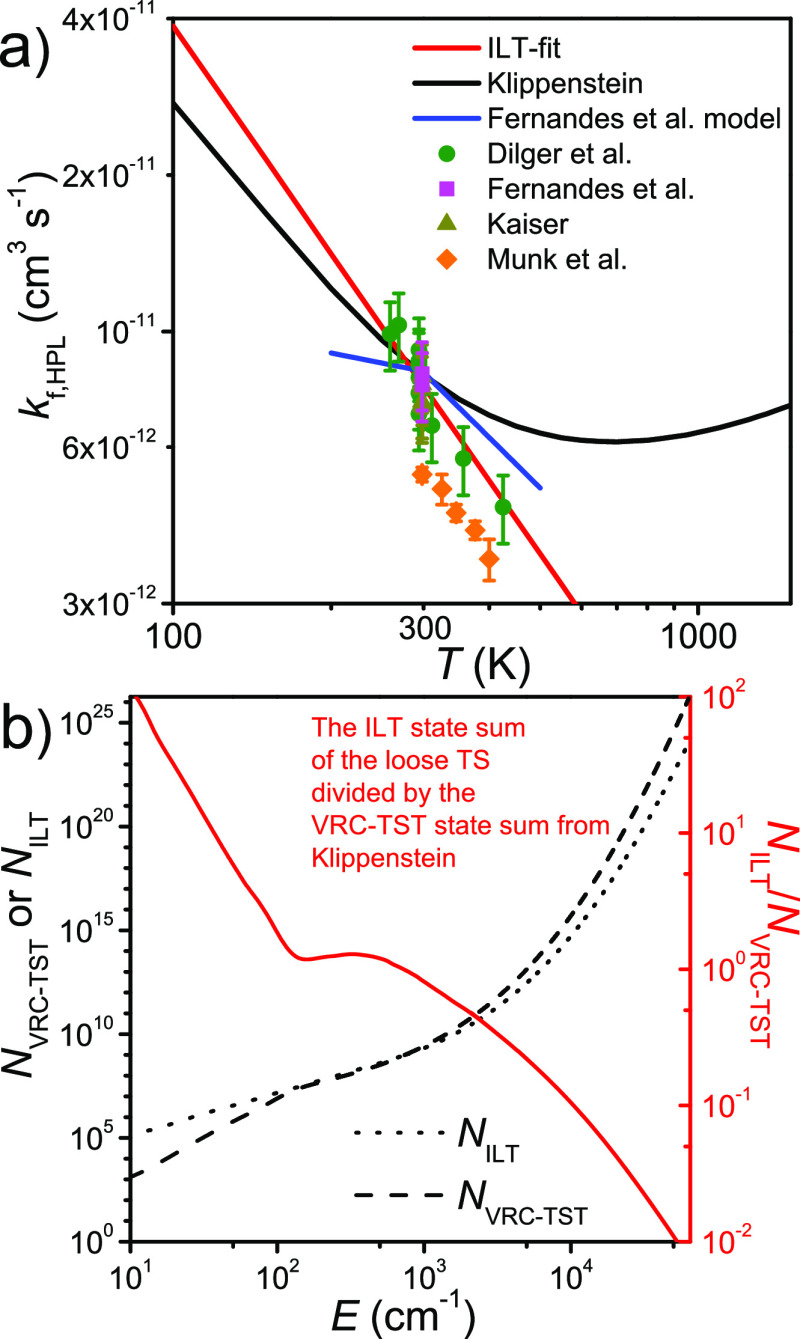
(a) High-pressure (canonical) C_2_H_5_^•^ + O_2_ → C_2_H_5_O_2_^•^ rate coefficients predicted by Klippenstein and the ILT-fit plotted as functions of temperature. The results are shown together with existing high-pressure measurements^6,17−19^ and the modeling results of Fernandes et al.^20^ (b) VRC-TST and ILT state sums (and their difference) plotted as functions of energy.^6^

Figure 6a displays the high-pressure limit (canonical) recombination rate coefficient calculated by Klippenstein^6^ and the one predicted by our ILT-fit as a function of temperature. These are shown together with the available high-pressure experimental measurements and the modeling results of Fernandes et al.^6,17−20^ For comparison purposes, we also show the difference between the VRC-TST and ILT state sums as a function of energy (Figure 6b). The canonical rate coefficient of Klippenstein is in good agreement with the ILT-fit one at room temperature (8.3 × 10^–12^ and 7.9 × 10^–12^ cm^3^ s^–1^, respectively. As temperature is decreased, the rate coefficients begin to diverge, but the agreement it still quite good at 100 K (28 × 10^–12^ cm^3^ s^–1^ and 39 × 10^–12^ cm^3^ s^–1^, respectively). The agreement at elevated temperature is much worse: Klippenstein predicts that the temperature dependence shifts from negative to positive at ∼700 K, whereas the ILT-fit predicts a constant negative temperature dependence. Since the ethyl + O_2_ system is quite small, one can expect Klippenstein’s VRC-TST calculations to be accurate and predict correctly the change in temperature dependence. Note that since we inverted only a single Arrhenius expression (with the exponential term set to zero) in the ILT-fit, the expression is unable to predict a change in temperature dependence. We tried using a sum of two Arrhenius expressions, but the output of the fit was essentially a single Arrhenius expression (the fitted temperature exponent was the same for both expressions). This failure is not entirely unexpected, as there are a limited number of high-pressure measurements, all within a relatively narrow temperature range (260–425 K). Although the ILT-fit better captures the high-pressure experimental data, we believe the canonical rate coefficient calculated by Klippenstein is more reliable over an extended temperature range (100–2000 K). Thus, we opted to use the *N*_VRC-TST_-fit model in the rest of our simulations.

**Figure 7:**
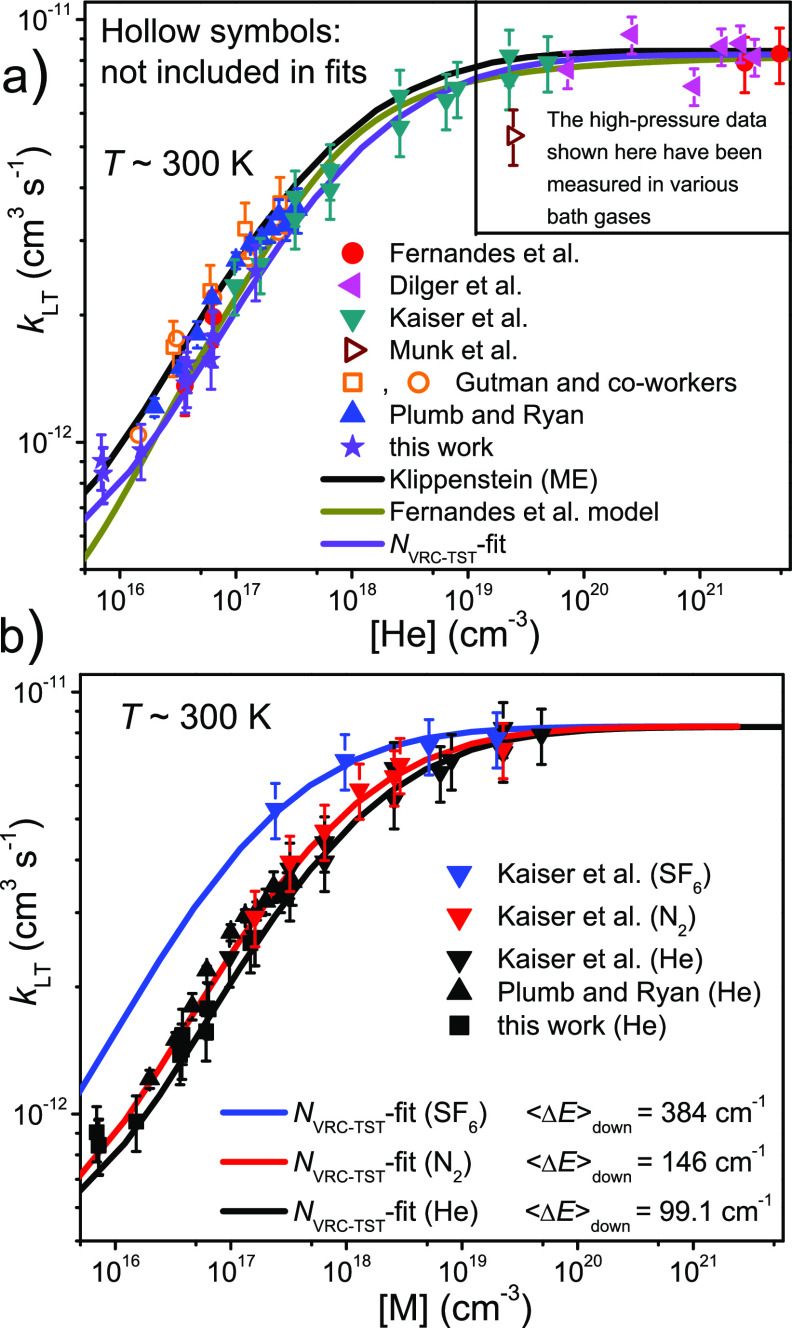
(a) Room-temperature falloff curve predicted by our optimized model compared to existing experimental measurements^9,11,15,17−20^ and the computational and modeling results of Klippenstein^6^ and Fernandes et al.^20^ (b) Room-temperature falloff curves in different bath gases predicted by our optimized model compared to existing experimental measurements.

**Figure 8:**
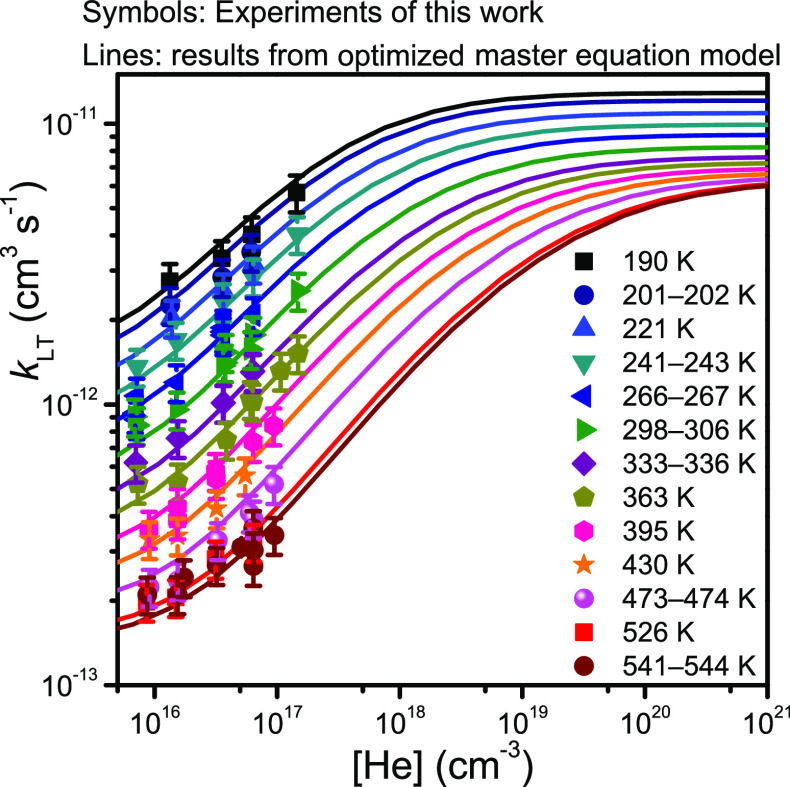
Falloff curves predicted by our optimized master equation model at different temperatures. The curves are shown together with the experimental results from this work.

The *N*_VRC-TST_-fit yielded ⟨Δ*E*⟩_down,300K_ = 99.1 cm^–1^ for helium bath gas data. This value may seem very low given that the model calculations by Jasper and Miller predicted 117 cm^–1^ for the smaller CH_3_^•^ + H^•^ (+ He) ⇌ CH_4_ (+ He) system.^36^ However, one-dimensional ME treatments are known to overestimate rate coefficients in the falloff region due to the neglect of angular momentum effects.^37^ One can compensate for this by using an artificially low ⟨Δ*E*⟩_down_. Thus, the value we obtain for ⟨Δ*E*⟩_down,300K_ may simply be low because we forced a one-dimensional model onto two-dimensional data. Klippenstein used a larger value, 180 cm^–1^, in his recent (one-dimensional) ME study of the C_2_H_5_^•^ + O_2_ reaction, and we compare his and our predictions for the room-temperature falloff curve in Figure 7a. The larger value he used is more consistent with the data of Plumb and Ryan^9^ and Gutman and co-workers,^11^,^15^ whereas our smaller value better reproduces the measurements in this work and those of Kaiser et al.^17^ The small value we obtained also does a fair job at reproducing the experimental falloff behavior at different temperatures (see Figure 8).

Kaiser et al. also performed some measurements in nitrogen and sulfur hexafluoride bath gases, and we used their results to obtain ⟨Δ*E*⟩_down,300K_ for N_2_ and SF_6_.^17^ These fits were performed so that all of the other parameters were fixed to the values obtained from the helium bath gas *N*_VRC-TST_-fit. The optimized values are reported in Table 2. Figure 7b shows room-temperature falloff curves in the different bath gases together with the existing experimental data.

### Equilibrium Constant

**Figure 9:**
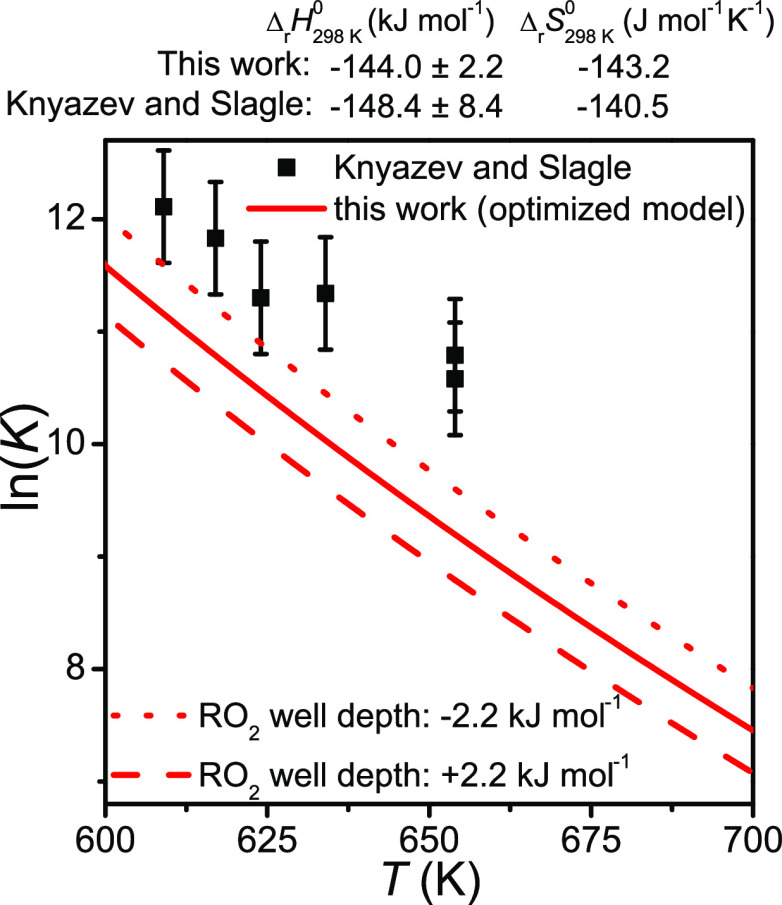
C_2_H_5_^•^ + O_2_ ⇌ C_2_H_5_O_2_^•^ equilibrium constant computed with our optimized model compared with the values reported by Knyazev and Slagle.^14^

Slagle et al. measured the equilibrium constant for the C_2_H_5_^•^ + O_2_ ⇌ C_2_H_5_O_2_^•^ reaction.^13^ Knyazev and Slagle later reanalyzed the data with an improved kinetic model that included an irreversible unimolecular loss channel for C_2_H_5_O_2_^•^. Unfortunately, even this improved mechanism is deficient because it considers only reaction R1 and the wall rates of C_2_H_5_^•^ and C_2_H_5_O_2_^•^; reaction R2 is not included. Ignoring reaction R2 is not justified under the low-pressure conditions of their experiments, which means that the formulas they use to determine the rate coefficients are not the appropriate ones. However, the form of their double-exponential fitting function is correct, with the exponential parameters corresponding to the CSEs λ_1_ and λ_2_ in the simplified ME model. Because MESMER allows the user to optimize parameters against experimental eigenvalues, we were able to use their exponential parameters in the parameter optimizations. In Figure 9 we compare the equilibrium constant computed with our optimized model with the values reported by Knyazev and Slagle. There is clear disagreement. When we simulated the reaction under the conditions of their measurements, we found that the well-skipping rate coefficient *k*_ws_ is roughly equal to the recombination rate coefficient *k*_f_. Omission of reaction R2 in the kinetic scheme leads to an overestimation of *k*_f_, which in turn results in an overestimation of the equilibrium constant.

### Ethene + Hydroperoxyl Yield

**Figure 10:**
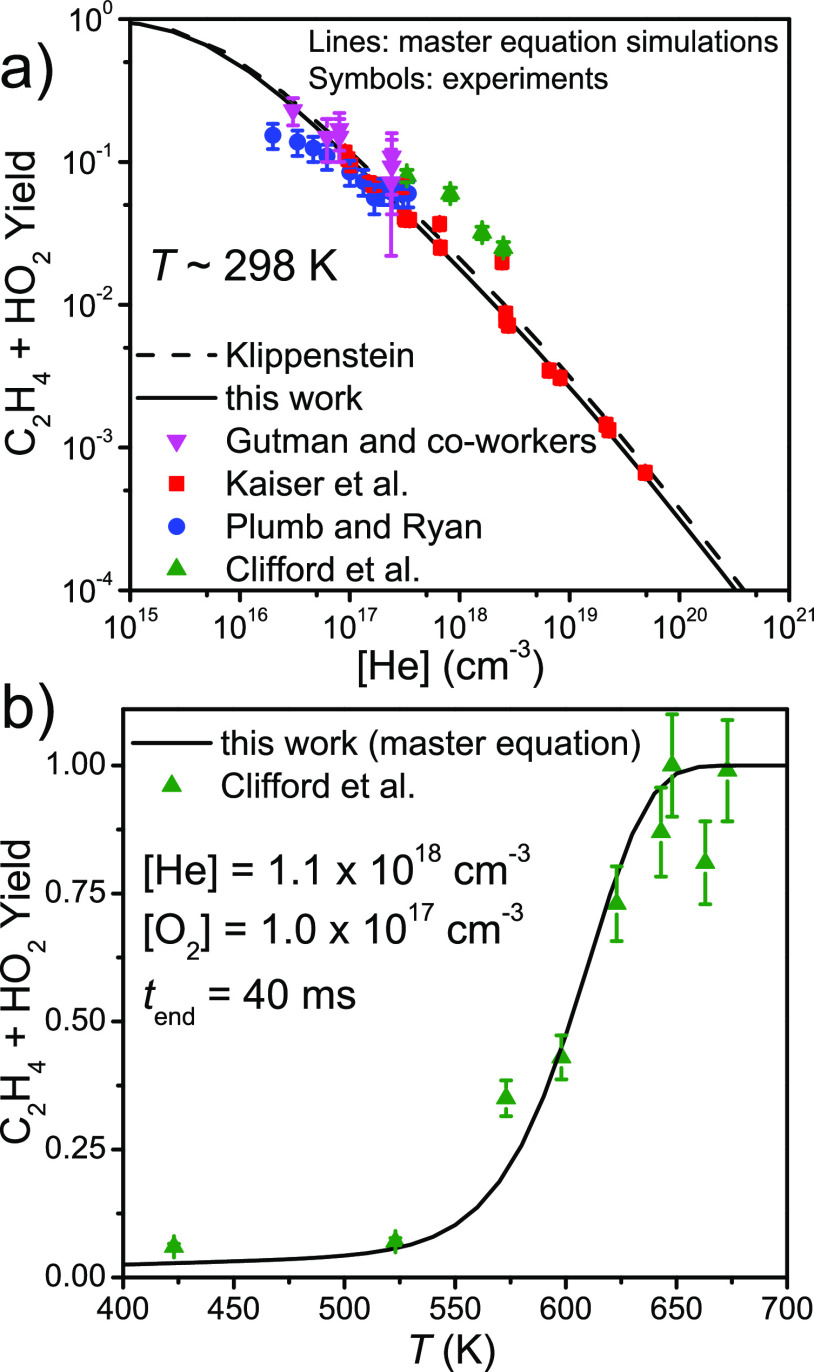
Simulated yield of C_2_H_4_ + HO_2_^•^ (a) as a function of pressure at room temperature and (b) as a function of temperature at [He] = 1.1 × 10^18^ cm^–3^. The results are compared to the measurements of Plumb and Ryan, Gutman and co-workers, Kaiser et al., and Clifford et al. and the master equation simulations of Klippenstein.^5,6,9−11^

Several authors^5,9−11^ have measured the ethene + hydroperoxyl yield of the title reaction, and these experiments provide yet another test for our ME model. In Figure 10 we compare the measured yields to the ones produced by our model and the model of Klippenstein.^6^ The O_2_ concentration was set to 1.0 × 10^17^ cm^–3^ and the yield time to *t* = 40 ms. Note that the comparison with the temperature-dependent values of Clifford et al.^5^ is somewhat crude, as the [O_2_] and termination time of their experiments are not exactly the same as those set in the simulations. At high temperatures, the prompt formation of C_2_H_4_ + HO_2_^•^ is followed by a slower formation reaction that originates from the peroxyl adduct either dissociating back to reactants (which is then followed by a well-skipping reaction to products) or directly dissociating to products. Because of the slower formation, the termination time of the experiments/simulations will have an effect on the C_2_H_4_ + HO_2_^•^ yield (unless the reaction is monitored for so long that the yield becomes 1). Despite these complications, the agreement between our model and the temperature-dependent yields of Clifford et al. is very good. The current results are also in good agreement at room temperature with the results of Klippenstein, Plumb and Ryan, Gutman and co-workers, and Kaiser et al.

### Eigenvalues and Rate Coefficients

**Figure 11:**
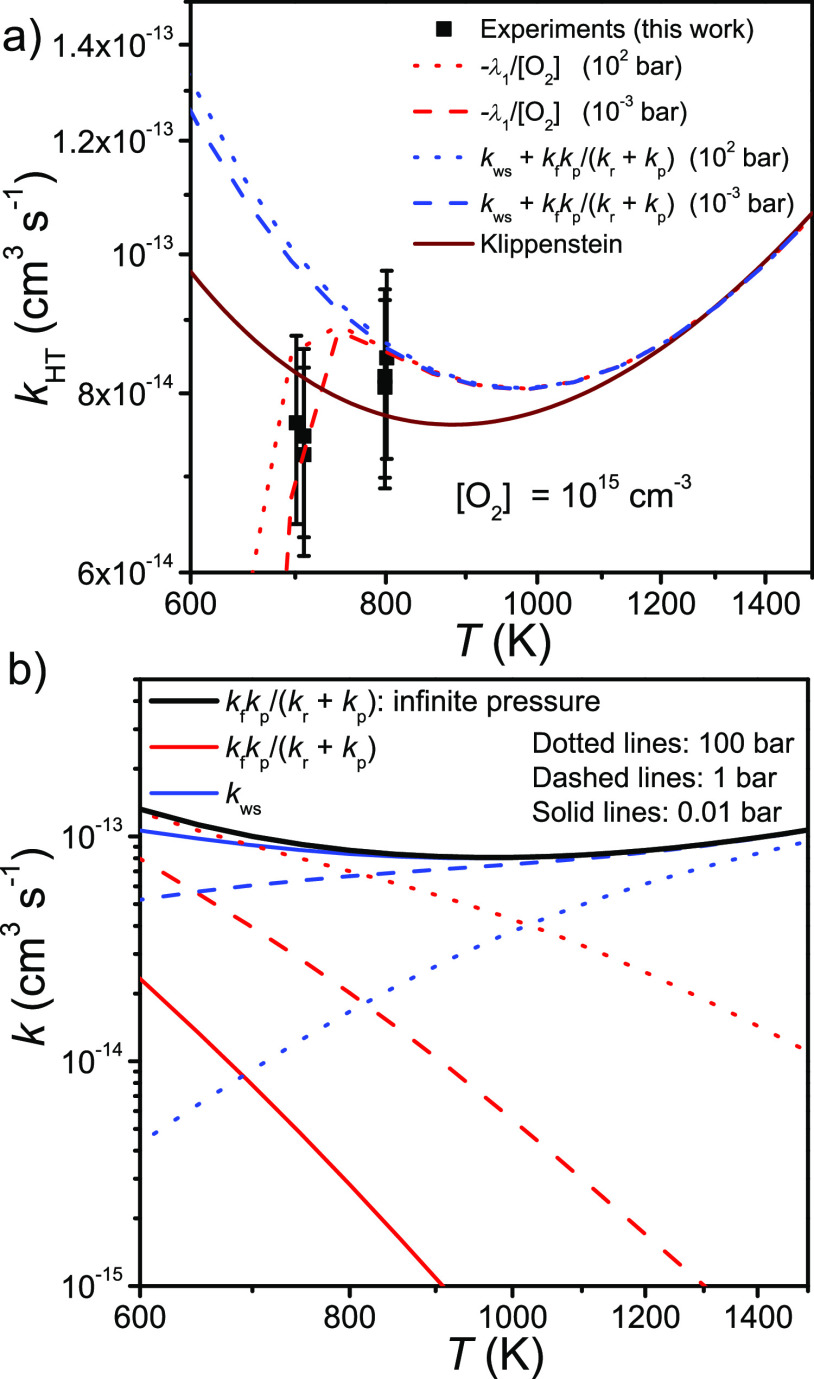
(a) Simulated high-temperature rate coefficient expressed in terms of λ_1_ or Bartis–Widom rate coefficients. The results are shown together with the experimental results and the prediction of Klippenstein.^6^ (b) Sequential mechanism (reaction R1) and well-skipping mechanism (reaction R2) rate coefficients plotted as functions of temperature at different pressures.

We mentioned in the Introduction that the high-temperature rate coefficient can be associated with λ_1_ but also that it can be expressed in terms of elementary rate coefficients if the pre-equilibrium approximation is made. In Figure 11a we show that these two approaches yield equivalent results when pre-equilibrium conditions apply. The elementary rate coefficients are obtained from Bartis–Widom analysis.^38,39^ Also shown in the figure are the high-temperature measurements of this work and Klippenstein’s prediction for the well-skipping rate coefficient at 10^–4^ bar.^6^ At such a low pressure the well-skipping rate coefficient can be equated with the total rate coefficient, so the comparison to the present results is valid. The small disagreement between the simulated rate coefficients is due to the CETS being about 1 kJ mol^–1^ lower in energy in our model. Both models agree with the current measurements within experimental uncertainty. The λ_1_ eigenvalue curves in Figure 11a indicate that at ∼700 K our measurements are not yet fully out of the transitional regime. This adds some ambiguity to the experimental results at ∼700 K because at this temperature a “good” rate coefficient might not yet exist.

In Figure 11b we illustrate how the relative importance of the sequential and well-skipping channels changes as pressure is increased or decreased. As expected, the importance of the well-skipping channel increases with decreasing pressure, while the opposite is true for the sequential channel. Although both of these channels are pressure-dependent, when one increases, there will be a compensating decrease in the other, and as a result, the total rate coefficient remains essentially the same.

**Figure 12:**
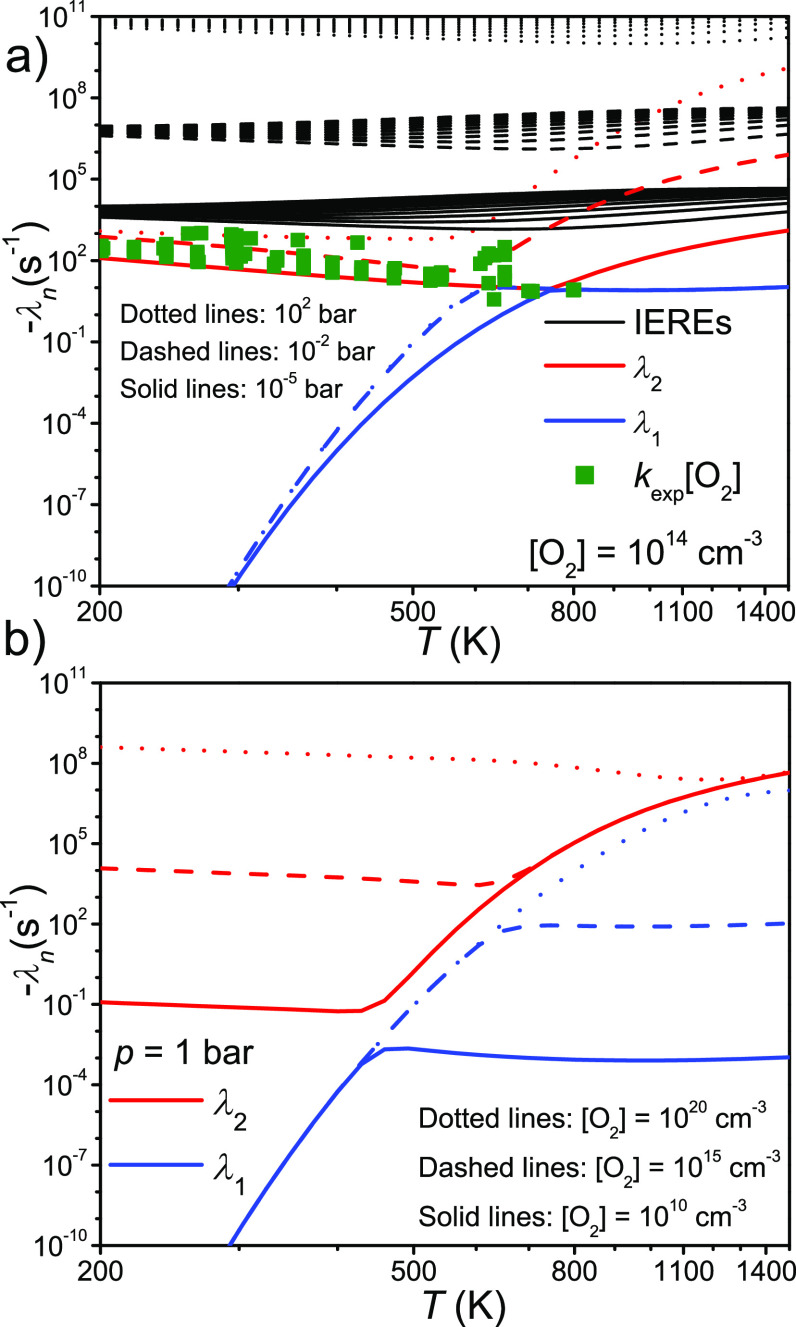
(a) Eigenvalue curves of the C_2_H_5_^•^ + O_2_ system plotted as functions of temperature at different pressures and constant [O_2_]. The simulation results are shown together with experimental data points measured at various temperatures between 3 × 10^–4^ bar and 2 × 10^2^ bar. (b) Eigenvalue curves of the C_2_H_5_^•^ + O_2_ system plotted as functions of temperature at different O_2_ concentrations and constant pressure.

Figure 12a demonstrates how the rate coefficient “jumps” from eigenvalue curve λ_2_ to λ_1_ as the high-temperature regime is entered. To show this clearly, we have plotted the experimental rate coefficient determinations (or *k*[O_2_] to be exact) together with simulated eigenvalue curves. The experimental data points shown are those that were included in the parameter optimization. It can also be clearly seen from this figure that λ_1_ is pressure-independent in the high-temperature regime for all practical purposes. Interestingly, λ_2_ does not merge with the continuum of internal energy relaxation eigenvalues (IEREs) even at relatively high temperatures (∼1500 K). This is true both at low and high pressures. Thus, Bartis–Widom analysis should yield reliable rate coefficients over very wide temperature and pressure ranges. Figure 12b depicts how varying [O_2_] changes the temperature ranges of the low- and high-temperature regimes. When [O_2_] = 10^20^ cm^–3^ and *p* = 1 bar, the reaction system is still in the “low-temperature” regime even at 1000 K.

We provide the temperature- and pressure-dependent Bartis–Widom rate coefficients in PLOG format in the Supporting Information to facilitate the use of the current results in atmospheric and combustion modeling. These results were simulated in N_2_ bath gas. The input file of our master equation model is also given.

## Conclusions

We have presented a comprehensive experimental and master equation study of the C_2_H_5_^•^ + O_2_ reaction. A motivation for the study was to check whether the rate coefficient measured by Gutman and co-workers^11,15^ is “too high”, as suggested by Fernandes et al.^20^ The rate coefficient measured in this study is indeed smaller and consistent with the model of Fernandes et al. The results of Gutman and co-workers are 20% larger at room temperature and between 30% and 50% larger at temperatures above 470 K. The experimental work was combined with master equation modeling. We used the current experimental results and measurements by other authors to fix key parameters in the model, after which the model was able to reproduce existing rate coefficient and reaction yield data. We provide accurate rate coefficients for the conjugate-alkene channel in the ethyl + O_2_ reaction and expect the results to be of use in atmospheric and combustion chemistry modeling.

## Supporting Information Available

MESMER input file (C2H5+O2.xml) and modified Arrhenius representations of Bartis–Widom rate coefficients in PLOG format (PLOG.txt) (ZIP)
